# Supplementary material for: SAM homeostasis is regulated by CFIm-mediated splicing of MAT2A
Source: eLife. 2021 May 5;10:e64930. doi: 10.7554/eLife.64930 (PMC8139829; doi:10.7554/eLife.64930)
Supplement: Transparent reporting form [file elife-64930-transrepform.docx]

***eLife’s* transparent reporting form**

We encourage authors to provide detailed information *within their submission* to facilitate the interpretation and replication of experiments. Authors can upload supporting documentation to indicate the use of appropriate reporting guidelines for health-related research (see [EQUATOR Network](about:blank)), life science research (see the BioSharing Information Resource), or the [ARRIVE guidelines](about:blank) for reporting work involving animal research. Where applicable, authors should refer to any relevant reporting standards documents in this form.

If you have any questions, please consult our Journal Policies and/or contact us: [editorial@elifesciences.org](about:blank).

**Sample-size estimation**

- You should state whether an appropriate sample size was computed when the study was being designed
- You should state the statistical method of sample size computation and any required assumptions
- If no explicit power analysis was used, you should describe how you decided what sample (replicate) size (number) to use

Please outline where this information can be found within the submission (e.g., sections or figure legends), or explain why this information doesn’t apply to your submission:

In this study, all statistical analyses were applied to gene expression or PAC-seq data, so replicate number is the critical parameter, not sample size. Replicates are addressed below.

**Replicates**

- You should report how often each experiment was performed
- You should include a definition of biological versus technical replication
- The data obtained should be provided and sufficient information should be provided to indicate the number of independent biological and/or technical replicates
- If you encountered any outliers, you should describe how these were handled
- Criteria for exclusion/inclusion of data should be clearly stated
- High-throughput sequence data should be uploaded before submission, with a private link for reviewers provided (these are available from both GEO and ArrayExpress)

Please outline where this information can be found within the submission (e.g., sections or figure legends), or explain why this information doesn’t apply to your submission:

Each quantified experiment was performed with a minimum of three biological replicates. In some cases, the experiments were more variable (e.g. intracellular SAM determination), so more replicates were performed to increase confidence and statistical power in the results.

A biological replicate is defined as cells that were treated (e.g. Met depletion, transfection, siRNA knockdown, etc.) on independent days with independent “splits” of the cells. Therefore, the replicates were either a different passage or, in some cases, cells that had been thawed at a different time.

The replicate numbers are given in each figure legend and independent replicate points are shown on each of the bar graphs in addition to error bars. Statistical methods are also listed in the figure legends.

There was no exclusion of outliers.

GEO submission has been made for our PAC-seq data (GSE158591) and the secure token for reviewer access has been included in the submitted paper (ojylsqokndihbyn).

**Statistical reporting**

- Statistical analysis methods should be described and justified
- Raw data should be presented in figures whenever informative to do so (typically when N per group is less than 10)
- For each experiment, you should identify the statistical tests used, exact values of N, definitions of center, methods of multiple test correction, and dispersion and precision measures (e.g., mean, median, SD, SEM, confidence intervals; and, for the major substantive results, a measure of effect size (e.g., Pearson's r, Cohen's d)
- Report exact p-values wherever possible alongside the summary statistics and 95% confidence intervals. These should be reported for all key questions and not only when the p-value is less than 0.05.

Please outline where this information can be found within the submission (e.g., sections or figure legends), or explain why this information doesn’t apply to your submission:

The replicate numbers are given in each figure legend and independent replicate data points are shown on each of the bar graphs in addition to error bars. Statistical methods are also listed in the figure legends and the fact that data are represented as mean±SD is also addressed in the figure legends.

In most cases, we used Student’s t-tests to compare between our samples as is standard in gene expression studies. In some figures, different comparisons were made among the samples and we tried to outline this clearly in the figure legends and/or the figure itself (e.g. Fig 3C). Statistical analysis for Venn diagram overlap used the SuperExact Test (Wang et al., 2015), which was specifically designed for this purpose.

(For large datasets, or papers with a very large number of statistical tests, you may upload a single table file with tests, Ns, etc., with reference to sections in the manuscript.)

**Group allocation**

- Indicate how samples were allocated into experimental groups (in the case of clinical studies, please specify allocation to treatment method); if randomization was used, please also state if restricted randomization was applied
- Indicate if masking was used during group allocation, data collection and/or data analysis

Please outline where this information can be found within the submission (e.g., sections or figure legends), or explain why this information doesn’t apply to your submission:

This is not applicable as we do not do any group allocation.

**Additional data files (“source data”)**

- We encourage you to upload relevant additional data files, such as numerical data that are represented as a graph in a figure, or as a summary table
- Where provided, these should be in the most useful format, and they can be uploaded as “Source data” files linked to a main figure or table
- Include model definition files including the full list of parameters used
- Include code used for data analysis (e.g., R, MatLab)
- Avoid stating that data files are “available upon request”

Please indicate the figures or tables for which source data files have been provided:

The PAC-seq and MAGeCK analyses include summary tables (Supplemental). The GEO submission for the PAC-seq data includes “source data” file as well as raw data.
